# Supplementary material for: Pathophysiological Significance of Neutrophilic Transfer RNA-Derived Small RNAs in Asymptomatic Moyamoya Disease
Source: Cells. 2021 May 1;10(5):1086. doi: 10.3390/cells10051086 (PMC8147334; doi:10.3390/cells10051086)

2D Structure	Local AU	Position

```
268              8mer-1a   297
 5'-tgaAACCCTATGAATG-TAAGCAATGTGGGa-3' utr
    ||  ||||   |  :|  ||  :|  ||||||
 3'-uccUUAGGAU--UGGCGAUCUGGUACACCCu-5' tRF
                   16151413   7 6 5 4 3 2
                3' Pairing     Seed
```

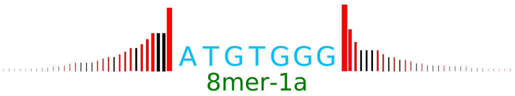

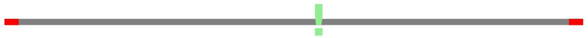

2D Structure	Local AU	Position

```
125             8mer-1a    154
 5'-agaAACCCTATCAATGTAAGCAGTGTGGGa-3' utr
     ||  ||||  |   :   ||  :|  :||||||
 3'-uccUUAGGAUUGGCG-AUCUGGUACACCCu-5' tRF
                 1615  1413    7 6 5 4 3 2
              3' Pairing       Seed
```

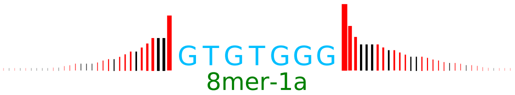

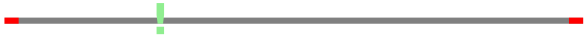

Supplement: Supplementary file 1 [file cells-10-01086-s001.zip › cells-1149161 3rd supplementary materials/Data Supplements/Data Supplement V.zip!/Data supplement 6-mRNAs targets of tsRNAs/tsRNA-Glu-TTC-010/Diagrams/tRF-Glu-TTC-010_vs_uc010xme.3(AC008770.2).pdf]
